# High tissue factor (CD142) expression identifies a poor-prognosis subgroup of esophageal and esophagogastric junction adenocarcinoma

**DOI:** 10.1186/s12885-026-17336-w

**Published:** 2026-11-10

**Authors:** Tillmann Bedau, Thomas Zander, Hans Schlößer, Reinhard Büttner, Christiane Bruns, Alexander Quaas

**Affiliations:** 1 Institute of Pathology, Faculty of Medicine, University Hospital Cologne, Cologne, Germany; 2 Department of Internal Medicine, Faculty of Medicine, University Hospital Cologne, Cologne, Germany; 3 Department of General, Visceral and Cancer Surgery, Faculty of Medicine, University Hospital of Cologne, Cologne, Germany

**Keywords:** Tissue Factor, CD142, Esophageal adenocarcinoma, Esophagogastric junction adenocarcinoma, Antibody–drug conjugate (ADC), Tisotumab vedotin, Prognostic biomarker

## Abstract

**Background:**

Tissue Factor (TF/CD142) is an emerging antibody–drug conjugate (ADC) target, but its prevalence, prognostic value and molecular context in adenocarcinoma of the esophagus (EAC) and esophagogastric junction (EGJ) remain uncharacterized.

**Methods:**

TF immunohistochemistry was scored by H-score (0–300) on a tissue microarray of 1,120 resected EAC/EGJ adenocarcinomas (1996–2020); the 1,006 assessable primary tumors formed the analysis cohort. Tumors were graded TF-negative (≤ 10), TF-low (11–100) or TF-high (> 100) and related to clinicopathologic features, tissue compartment, neoadjuvant therapy, HER2, Claudin 18.2 (CLDN18.2), MET, mismatch-repair (MMR) status and overall survival. Primary tumors and lymph-node metastases were compared within patients.

**Results:**

TF was positive in 486 of 1,006 tumors (48.3%) and high in 225 (22.4%). TF-positive tumors were more often pT3–4 (*p* < 0.001) and node-positive (*p* = 0.002), and TF grade agreed moderately between paired primary tumors and lymph-node metastases (*n* = 247; κ 0.57 at H-score > 100) without a systematic shift (*p* = 0.91). TF was independent of HER2 (*p* = 0.80) and MMR status (*p* = 0.14), inversely associated with CLDN18.2 (TF-high vs. TF-negative: 12.8% vs. 30.2%; *p* < 0.001) and positively with MET overexpression (8.6% vs. 1.0%; *p* < 0.001). Over a median follow-up of 67 months (749 patients, 428 deaths), the survival detriment was confined to TF-high tumors (median overall survival 19.3 vs. 36.8 and 42.2 months; *p* < 0.001). Adjusted for age, pT, nodal status and neoadjuvant therapy, TF-high remained adverse (HR 1.67, 95% CI 1.32–2.11; *p* < 0.001) whereas TF-low did not (HR 0.97; *p* = 0.83). The TF-high association held with TF as a continuous variable (HR 1.11 per 50 points; *p* < 0.001) and after adjustment for treatment era, margin, grade and lymphovascular invasion. TF positivity was similar in treatment-naïve and in residual post-neoadjuvant tumors (*p* = 0.89), but TF-high concentrated in poorly regressed tumors (29.2% vs. 11.6%; trend *p* < 0.001).

**Conclusions:**

TF is expressed in nearly half of EAC/EGJ adenocarcinomas and at high level in about one quarter, where it is independently associated with shorter overall survival. TF expression remains prevalent in residual tumors after neoadjuvant therapy and marks a compartment distinct from HER2 and CLDN18.2 and enriched for MET. TF-high is a candidate biomarker for prospective trials of TF-directed ADCs, in which its predictive value and the H-score > 100 threshold need testing.

**Supplementary Information:**

The online version contains supplementary material available at https://doi.org/10.1186/s12885-026-17336-w.

## Introduction

Esophageal adenocarcinoma (EAC) and adenocarcinoma of the esophagogastric junction (EGJ) represent clinically challenging malignancies with a steadily increasing incidence in Western countries. In Western Europe, the rising burden of EAC has been linked to the increasing prevalence of gastroesophageal reflux disease, Barrett’s esophagus, obesity, and metabolic risk factors [1, 2]. Despite substantial advances in multimodal treatment strategies, the prognosis of EAC remains poor. In patients with resectable disease, perioperative chemotherapy with fluorouracil, leucovorin, oxaliplatin and docetaxel (FLOT) is the established backbone for gastric and EGJ adenocarcinoma [3] and, since the phase 3 ESOPEC trial, for esophageal adenocarcinoma [4]. The phase 3 MATTERHORN trial has since shown that adding the PD-L1 inhibitor durvalumab to perioperative FLOT prolongs event-free and overall survival, setting a new perioperative standard for resectable gastric and EGJ adenocarcinoma [5]. Nevertheless, disease recurrence remains common and the prognosis of patients with recurrent or metastatic EAC remains unfavorable. Additional treatment options, and the biomarkers to direct them, are needed.

In advanced or metastatic gastroesophageal adenocarcinoma, several tissue-based biomarkers are now routinely assessed to guide treatment selection. HER2 overexpression or amplification identifies patients eligible for HER2-directed therapy, including trastuzumab and the antibody–drug conjugate (ADC) trastuzumab deruxtecan [6–8]. PD-L1 expression is used to support the use of immune checkpoint inhibition, while deficient mismatch repair or microsatellite instability-high status defines a biologically distinct subgroup with pronounced sensitivity to immunotherapy [9–11]. More recently, Claudin 18.2 (CLDN18.2) has emerged as a clinically validated therapeutic target in gastric and EGJ adenocarcinoma, with the anti-CLDN18.2 antibody zolbetuximab improving outcomes in CLDN18.2-positive, HER2-negative disease [12, 13]. Importantly, these biomarkers are determined directly on tumor tissue, predominantly by immunohistochemistry, in situ hybridization and/or molecular assays, underscoring the central role of tissue-based biomarker assessment in precision oncology for EAC.

However, currently actionable biomarkers identify only partly overlapping patient subgroups, and many patients still lack effective targeted treatment options. Antibody–drug conjugates have become a particularly important therapeutic modality in this context. ADCs combine antigen-specific tumor cell binding with delivery of highly potent cytotoxic payloads and may therefore exploit cell-surface antigens even when these are not classical oncogenic drivers. The clinical success of trastuzumab deruxtecan in HER2-positive gastric and EGJ adenocarcinoma has established ADC therapy as a clinically relevant principle in upper gastrointestinal cancer [8]. In parallel, CLDN18.2-directed ADCs are currently under clinical investigation and have shown encouraging early activity in advanced gastric and EGJ adenocarcinoma [14].

Tissue Factor (TF; CD142; coagulation factor III) is an emerging ADC target of particular translational interest. TF is a transmembrane glycoprotein and the principal initiator of the extrinsic coagulation cascade through binding of factor VII/VIIa. Beyond hemostasis, TF contributes to tumor progression by promoting tumor-associated coagulation, angiogenesis, invasion, metastatic dissemination and intracellular signaling through protease-activated receptor pathways [15, 16]. Aberrant TF expression has been reported in a broad spectrum of solid tumors and has frequently been associated with aggressive tumor biology and poor clinical outcome. From a therapeutic perspective, TF is attractive because it is a membrane-bound, antibody-accessible antigen that can be assessed by immunohistochemistry in formalin-fixed paraffin-embedded tumor tissue, analogous to established biomarkers such as HER2, PD-L1 and CLDN18.2. Preclinical data provide strong biological support for TF-directed ADC therapy.

Breij et al. developed TF-specific ADCs and demonstrated potent cytotoxic activity in vitro and in vivo that was dependent on TF expression. The lead construct TF-011-MMAE/HuMax-TF-ADC induced marked antitumor activity across patient-derived xenograft models from several solid tumor types, including models with heterogeneous TF expression, supporting TF membrane expression as a functional mediator of ADC internalization and payload delivery [17]. High TF turnover and efficient intracellular trafficking have further been proposed as mechanisms facilitating ADC-based targeting [18].

Clinical development has advanced most prominently with tisotumab vedotin, a TF-directed ADC composed of an anti-TF antibody conjugated to monomethyl auristatin E. The confirmatory phase 3 innovaTV 301 trial showed an overall-survival benefit in recurrent or metastatic cervical cancer, and tisotumab vedotin now has full approval for this indication in the United States and the European Union [19–21].

Activity in gastrointestinal tumors, however, is barely mapped: the first-in-human basket study included a small, histologically mixed esophageal cohort but reported no esophageal-specific response rate [22], and the newer TF-ADC MRG004A has shown its clearest signal in pancreatic cancer, where responses concentrated in tumors with high TF expression [23]. A second generation of TF-ADCs carrying topoisomerase-1 payloads has since entered phase 1 testing (NCT06597721), while an earlier auristatin-based construct (XB002) was discontinued in 2024. Against this activity, TF expression in EAC itself is essentially uncharacterized. The few prior “esophageal” series are either squamous [24] or of unspecified histology [25], and no large adenocarcinoma-specific analysis has been reported.

We therefore set out to characterize TF expression in a large, clinically and molecularly annotated cohort of EAC and EGJ adenocarcinomas – to our knowledge the largest study of TF in this disease. We asked how frequent TF expression is and how it is distributed across primary tumors and their nodal and distant metastases; whether it carries prognostic weight independent of stage; whether it remains prevalent in residual tumor after neoadjuvant therapy, the tissue from which a recurrence would arise; and how it relates to the established and emerging biomarkers HER2, CLDN18.2, MET and MMR/MSI status. If TF marks a large, prognostically distinct subgroup that current markers miss, that would strengthen the rationale for TF-directed therapy in EAC/EGJ.

## Materials and methods

### Study population and tissue samples

This retrospective cohort study included patients with histologically confirmed adenocarcinoma of the esophagus (EAC) and esophagogastric junction (EGJ) who underwent surgical resection at the University Hospital Cologne between 1996 and 2020. Patients were treated with right transthoracic esophagectomy and two-field lymphadenectomy (mediastinal and abdominal lymph nodes). Intestinal passage was reconstructed with a high intrathoracic esophagogastrostomy as described previously [26]. Neoadjuvant therapy consisted of either radiochemotherapy according to the Chemoradiotherapy for Oesophageal Cancer followed by Surgery (CROSS) protocol (carboplatin, paclitaxel, and intensity-modulated radiotherapy) or perioperative chemotherapy with fluorouracil, leucovorin, oxaliplatin, and docetaxel (FLOT). Patients whose neoadjuvant regimen was not classified as CROSS or FLOT in the follow-up database formed the group “other neoadjuvant therapy”. For analysis, neoadjuvant status was categorized as treatment-naïve, CROSS, FLOT, other neoadjuvant therapy, or unknown. During the first two years after surgery, clinical follow-up was performed every three months, followed by annual visits. Follow-up included review of the patient’s medical history, physical examination, abdominal ultrasound, chest radiography, and additional diagnostics when indicated. Overall survival was taken from the institutional follow-up database; cause of death was not recorded. Only resection specimens with residual tumor could be represented on the tissue microarray, so patients with a complete pathologic response (ypT0N0) were not part of the cohort.

### Tissue microarray construction

Tissue microarrays were generated from formalin-fixed, paraffin-embedded tumor specimens as reported previously [27]. Briefly, tissue cores 1.2 mm in diameter were transferred to the recipient paraffin block with a self-constructed, semi-automated precision instrument. Most patients were represented by one primary-tumor core (986 of 1,006) and 20 by two. Tonsil tissue was included as a control. Primary tumor, lymph-node metastasis, and distant-metastasis cores were represented on the arrays, allowing TF expression to be compared across tissue compartments.

### Tissue factor immunohistochemistry (TF, CD142)

Tissue Factor protein (CD142) expression was assessed by immunohistochemistry using an anti-TF antibody (Abcam, clone EPR22548-240, rabbit monoclonal; 1:1000 dilution, EDTA buffer) on a Leica Bond-Max staining platform (Leica Biosystems, Germany) according to the manufacturer’s instructions. Appropriate positive and negative controls were included in each staining run. Slides were scored by experienced pathologists blinded to clinical data. Membranous staining intensity was graded as 0 (no staining), 1+ (weak), 2+ (moderate), or 3+ (strong), and the percentage of tumor cells at each intensity was recorded. The H-score was calculated as (1 x % of 1 + cells) + (2 x % of 2 + cells) + (3 x % of 3 + cells), giving a value from 0 to 300. Tumors were classified as TF-positive at an H-score greater than 10 and TF-negative at an H-score of 10 or below. For the three-tier grade, tumors were classified as TF-negative (H-score 10 or below), TF-low (H-score 11 to 100), or TF-high (H-score above 100). When a patient contributed more than one assessable primary-tumor core, the highest-scoring core was used for patient-level analyses, and the mean of the cores was used in a sensitivity analysis. The same rule gave one primary-tumor and one metastasis score per patient for the paired comparison of tissue compartments. The TF-low/TF-high boundary was set at the median H-score of the TF-positive tumors (100; interquartile range 40 to 180), that is, a median split among expressing tumors. A median split of the whole cohort was not available because the cohort median H-score was 0. The boundary was therefore defined on the distribution of the marker alone, without reference to survival or to any other outcome, and its robustness was examined post hoc as described below.

### Additional biomarker assessment

Established and emerging therapeutic biomarkers were assessed on serial sections of the same cohort. HER2 status was determined by immunohistochemistry with reflex fluorescence in situ hybridization (FISH). HER2 IHC was graded from 0 to 3+; tumors were classified as HER2-positive when IHC was 3+, or when IHC was 2 + with *ERBB2* amplification on FISH. *ERBB2* amplification was defined as a HER2/CEN17 ratio of 2.0 or greater, or a mean HER2 copy number of 6.0 or more per cell in the presence of a ratio below 2.0; methodological details have been described previously [28]. IHC 2 + tumors without a FISH result were treated as equivocal and excluded from HER2 comparisons, and IHC 0 or 1 + tumors were classified as HER2-negative. Claudin 18.2 expression was assessed by immunohistochemistry using the VENTANA CLDN18 (43–14 A) assay (Roche Diagnostics, Basel, Switzerland) and scored as positive when 75% or more of tumor cells showed moderate (2+) or strong (3+) membranous staining, as previously reported [29]. MET (c-MET) protein expression was assessed by immunohistochemistry using the VENTANA MET (SP44) RxDx assay (Roche Diagnostics, Basel, Switzerland) and quantified with an H-score on the same 0 to 300 scale as TF; tumors were classified as MET-positive at an H-score of 150 or greater, reflecting strong overexpression, as previously described [30]. Mismatch repair (MMR) status was determined by immunohistochemistry as previously described [31]. Tumors with loss of MLH1 and PMS2 expression were classified as mismatch repair-deficient (dMMR), and tumors with retained expression as mismatch repair-proficient (pMMR).

### Clinical data collection

Clinical and pathological data were retrieved from electronic medical records and pathology reports, including age at operation, sex, year of operation, pathological TNM stage, resection margin, tumor grade, lymphatic and venous invasion, treatment history, and survival outcomes. Tumor location (esophagus versus EGJ, Siewert type) and pretreatment clinical stage were not available for analysis.

### Tumor regression grading

Resection specimens from pretreated patients were assessed for histopathologic tumor regression, quantified as the proportion of vital (residual) tumor cells remaining in the tumor bed: more than 50%, 10% to 50%, or less than 10%; specimens with no residual vital tumor were recorded separately. Regression assessment is not applicable to treatment-naïve resections and was available for 563 of the 1,006 patients in the analysis cohort. One patient with no residual vital tumor was excluded from the regression-stratified analyses, complete pathologic response leaving no tumor to represent on the tissue microarray.

### Statistical analysis

Descriptive statistics were used to summarize patient characteristics and TF expression. Unless otherwise specified, patient-level analyses used one primary-tumor core per patient (the highest-scoring assessable core), and primary tumors and metastases were compared within patients. Categorical variables were compared with Fisher’s exact test or the chi-square test, as appropriate. Continuous variables were compared with the Mann-Whitney U test (two groups) or the Kruskal-Wallis test (more than two groups). Overall survival was estimated by the Kaplan-Meier method, and differences between groups were assessed by the log-rank test. Multivariable Cox proportional hazards regression was used to evaluate the independent prognostic value of TF expression, adjusting for age (per 10 years), pT category (T3 to T4 versus T1 to T2), nodal status (node-positive versus node-negative), and neoadjuvant therapy (treated versus naïve). To test the robustness of the findings to threshold choice, the TF-positive fraction was recomputed across H-score cut-offs (greater than 0, 10, 50, and 100; Additional file 1: Figure S1), and the TF-high definition was varied across H-score cut-offs from greater than 10 to greater than 250 in steps of 10, at each of which the TF-high group was compared with all remaining patients by log-rank test and by Cox regression, unadjusted and adjusted for the same covariates as the primary model (Additional file 1: Figure S2, Table S1). Because the TF-high boundary had been fixed on the marker distribution rather than on outcome, the threshold maximizing the log-rank statistic is reported only as corroboration and no adjustment for the multiplicity of the candidate thresholds was applied. The covariates of the primary model were fixed before analysis as established prognostic factors recorded for nearly all patients. The proportional-hazards assumption was checked with Schoenfeld residuals. Patients with and without follow-up were compared with the chi-square or Mann-Whitney U test (Additional file 1: Figure S3, Table S2). Resection margin, tumor grade and lymphatic and venous invasion were recorded for 56% to 77% of the survival cohort and were added in complete-case sensitivity models. To separate the effect of these covariates from that of the smaller sample, the primary model was refitted in the same patients. Sex and treatment era (year of operation 1996–2005, 2006–2012 and 2013–2020, as a covariate or as a stratification variable) were added in models on all 730 patients. Further sensitivity models excluded patients with documented distant metastasis at resection (pM1), used the mean instead of the highest-scoring primary core, and were restricted to survival records matched by accession number (Additional file 1: Table S3). The TF H-score was also analyzed per 50 points and with restricted cubic splines with three and four knots at Harrell’s default percentiles of the H-score among TF-positive tumors. Non-linearity was tested by likelihood ratio against the linear term (Additional file 1: Figure S4). Because the three-tier boundaries were set on the observed distribution, the comparison of model fit by Akaike’s information criterion (AIC) is descriptive. Effect modification by pT, pN, combined pT/pN group, neoadjuvant therapy, regimen and treatment era was tested by likelihood ratio for a TF-high × subgroup interaction; these models compare TF-high with all other tumors (Additional file 1: Figure S5). Agreement between paired primary tumors and lymph-node metastases was measured with Cohen’s κ, unweighted for the H-score > 10 and > 100 dichotomies and linearly weighted for the three-tier grade, with percentile bootstrap 95% CIs from 10,000 resamples. Paired differences were tested with the exact McNemar test and with the Wilcoxon signed-rank test, retaining tied pairs (Pratt method). The difference in TF-high prevalence between FLOT and CROSS was examined by logistic regression adjusted for ypT, ypN, regression grade and year of operation, and in patients operated in 2010 or later. Trends in TF-high prevalence over calendar time were examined by logistic regression on year of operation. All tests were two-sided, and p-values below 0.05 were considered statistically significant. Analyses were performed in Python 3.12 with polars for data handling, SciPy for statistical testing, lifelines for Kaplan-Meier estimation, log-rank testing, and Cox proportional hazards regression, and statsmodels for logistic regression. Ordered categories were tested for trend with the Cochran–Armitage test, and the association between residual vital tumor and TF-high expression was additionally assessed by logistic regression adjusted for ypT and ypN category. No adjustment for multiple comparisons was made; the biomarker, neoadjuvant and regression analyses are exploratory and hypothesis-generating. Code for data visualization and for the additional analyses of the revision was written with the assistance of large language models (Claude Opus 4.8 and Claude Opus 5.5, Anthropic) and checked by the authors.

## Results

### Patient characteristics

Of 1,120 patients with esophageal adenocarcinoma (EAC) or adenocarcinoma of the esophagogastric junction (EGJ) who underwent tissue microarray-based analysis of Tissue Factor (TF) expression, 1,006 had an assessable primary-tumor TF H-score and formed the analysis cohort; the remaining 114 lacked an evaluable primary-tumor core. The cohort was predominantly male (87.9%), with a median age of 64 years (IQR 56–71). Most tumors presented as locally advanced disease: pT3 tumors accounted for 58.8% of cases, and nodal metastases were present in 60.2% of patients. Neoadjuvant treatment had been given to 55.1% of patients (CROSS in 28.8%, FLOT in 8.3% and other regimens in 18.0%), while 29.2% were treatment-naïve. Baseline clinicopathologic characteristics stratified by TF status (TF-positive, H-score > 10) are summarized in Table 1.

**Table 1:** Clinicopathologic characteristics of 1006 patients with histologically confirmed adenocarcinoma of the esophagus or esophagogastric junction and an assessable primary-tumor TF H-score (114 of 1120 unassessable), stratified by TF status (positive = H-score > 10)

Characteristic	Overall (*n* = 1006)	TF-negative (*n* = 520)	TF-positive (*n* = 486)	*p*
**Sex**,** n (%)**				0.77
Male	884 (87.9%)	455 (87.5%)	429 (88.3%)	
Female	120 (11.9%)	64 (12.3%)	56 (11.5%)	
Unknown	2 (0.2%)	1 (0.2%)	1 (0.2%)	
**Age at operation**,** median [IQR]**,** y**	64 [56–71]	64 [56–70]	65 [56–72]	0.11
Not recorded, n (%)	7 (0.7%)	3 (0.6%)	4 (0.8%)	
**pT category**,** n (%)**				< 0.001
T1	178 (17.7%)	116 (22.3%)	62 (12.8%)	
T2	190 (18.9%)	107 (20.6%)	83 (17.1%)	
T3	592 (58.8%)	272 (52.3%)	320 (65.8%)	
T4	30 (3.0%)	15 (2.9%)	15 (3.1%)	
Not assessable	16 (1.6%)	10 (1.9%)	6 (1.2%)	
**pN category**,** n (%)**				0.002
N0	374 (37.2%)	217 (41.7%)	157 (32.3%)	
N1	286 (28.4%)	149 (28.7%)	137 (28.2%)	
N2	166 (16.5%)	71 (13.7%)	95 (19.5%)	
N3	154 (15.3%)	68 (13.1%)	86 (17.7%)	
Not assessable	26 (2.6%)	15 (2.9%)	11 (2.3%)	
**Neoadjuvant therapy**,** n (%)**				0.49
Treatment-naïve	294 (29.2%)	152 (29.2%)	142 (29.2%)	
CROSS	290 (28.8%)	155 (29.8%)	135 (27.8%)	
FLOT	83 (8.3%)	37 (7.1%)	46 (9.5%)	
Other neoadjuvant	181 (18.0%)	98 (18.8%)	83 (17.1%)	
Unknown	158 (15.7%)	78 (15.0%)	80 (16.5%)	

Percentages are of the column total andinclude the unknown / not-assessable line, so each block sums to 100%. P-values: Fisher exact (sex), Mann–Whitney U (age), χ² (pT, pN, neoadjuvant);unknown and not-assessable categories are excluded from the tests. Within-category (row) percentages of the TF grade are given in Additional file 1: Table S4

TF-positive tumors were associated with more advanced local tumor growth and nodal spread. Compared with TF-negative tumors, TF-positive cases more often showed pT3 disease (65.8% vs. 52.3%), whereas early pT1 tumors were less frequent among TF-positive cases (12.8% vs. 22.3%; *p* < 0.001). Nodal involvement was likewise enriched in TF-positive tumors, with fewer node-negative cases (32.3% vs. 41.7%) and a higher frequency of N2–N3 disease (37.2% vs. 26.7%; *p* = 0.002). Within categories, the TF-positive rate rose from 34.8% in pT1 to 54.1% in pT3 tumors and from 42.0% in pN0 to 57.2% in pN2 tumors (Additional file 1: Table S4). TF status did not differ by sex (*p* = 0.77), age at surgery (*p* = 0.11) or neoadjuvant treatment group (*p* = 0.49); in pretreated patients, this comparison concerns residual tumor. TF-high tumors became more frequent with later year of operation (17.3% in 1996–2005, 20.2% in 2006–2012 and 24.4% in 2013–2020; OR 1.20 per 5 years, 95% CI 1.04–1.39; *p* = 0.011). Treatment-naïve tumors showed no such trend (OR 1.05; *p* = 0.69), so the rise probably reflects the growing share of pretreated, and in particular FLOT-treated, tumors.

### TF Expression Distribution and Grading

**Fig. 1:**
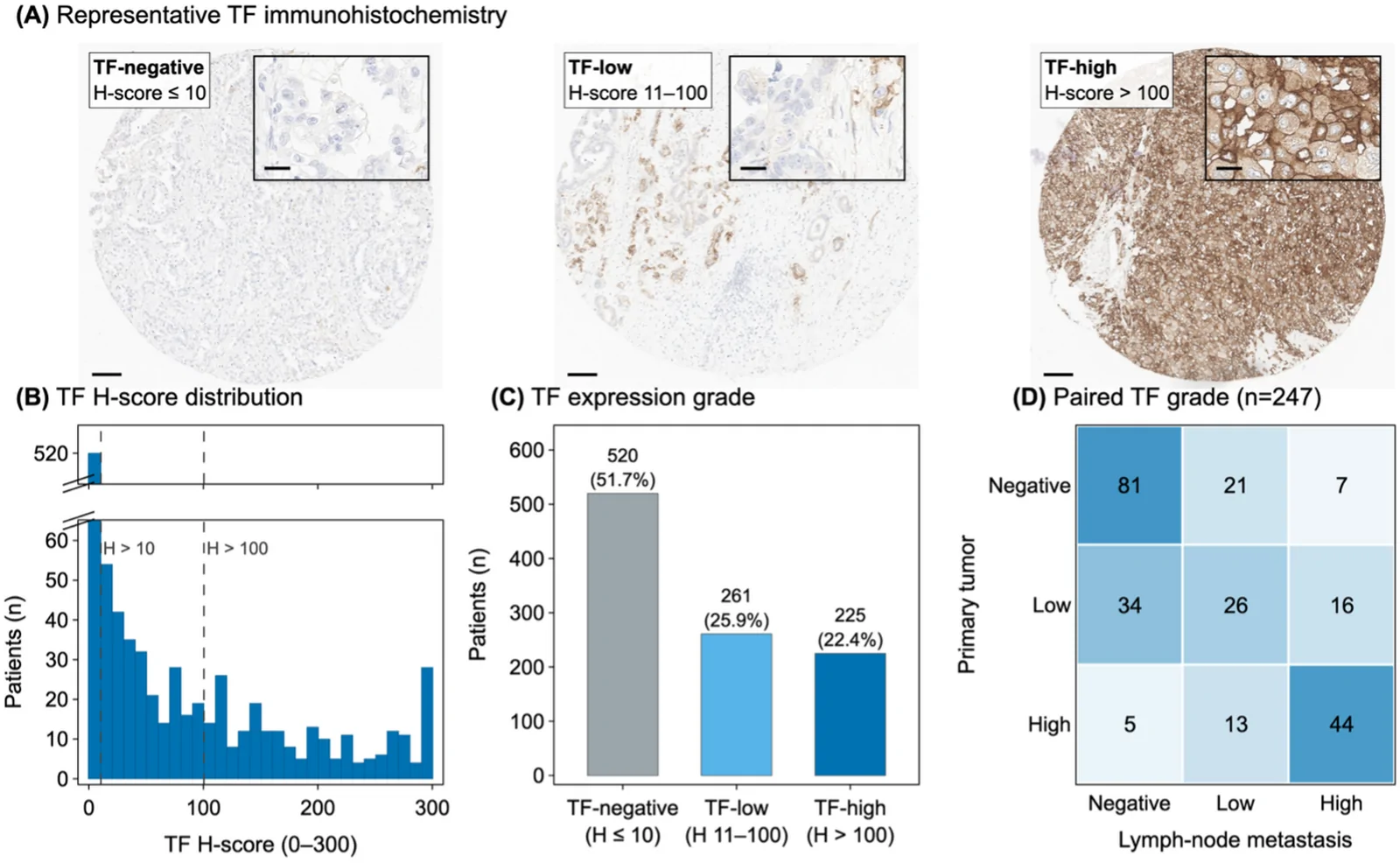
Tissue Factor (TF) expression in EAC. **A** Representative TF immunohistochemistry: whole tissue-microarray cores from a TF-negative (H-score ≤ 10), a TF-low (H-score 11–100) and a TF-high (H-score > 100) tumor, each with a detail inset (top right) at 40× original magnification. Scale bars, 100 μm (core overview) and 20 μm (inset); all three cores are shown at identical magnification. **B** Distribution of TF H-scores across 1,006 assessable primary tumors, plotted on a broken y-axis: the upper segment shows the TF-negative peak to its true height (*n* = 520) and the lower segment resolves the positive tail. Dashed lines mark the positivity threshold (H-score > 10) and the TF-low/TF-high boundary (H-score > 100). **C** Three-tier TF grade: TF-negative (H-score ≤ 10, *n* = 520, 51.7%), TF-low (11–100, *n* = 261, 25.9%), and TF-high (> 100, *n* = 225, 22.4%). **D** Three-tier TF grade of the primary tumor (rows) and the synchronous lymph-node metastasis (columns) in 247 patients with both (highest-scoring core of each). Cells give the number of patients; shading gives the share of the row. Agreement: κ 0.46 at H-score > 10, κ 0.57 at H-score > 100 and linearly weighted κ 0.51 for the three-tier grade; paired H-scores did not differ (Wilcoxon *p* = 0.91)

TF expression was quantified by an H-score ranging from 0 to 300, with representative staining for each grade shown in Fig. 1A. Among the 1,006 assessable primary tumors, 486 (48.3%) were TF-positive at the predefined threshold of H-score > 10, and 520 (51.7%) were TF-negative (Fig. 1B). Under the three-tier scheme, 520 tumors (51.7%) were TF-negative (H-score ≤ 10), 261 (25.9%) were TF-low (H-score 11–100), and 225 (22.4%) were TF-high (H-score > 100) (Fig. 1C). Close to half of all EAC/EGJ tumors therefore showed detectable TF expression, and roughly one-quarter reached high-level expression. The positive fraction was near-identical at the > 0 and > 10 cut-offs and fell to 32.1% at > 50 and to 22.4% at > 100 (Additional file 1: Figure S1). The TF-high boundary of > 100 was the median H-score of the 486 TF-positive tumors (IQR 40–180) and was thus fixed on the distribution of the marker alone; its consequences for the survival analysis are examined in Additional file 1: Figure S2 and Table S1.

### TF expression in paired primary tumors and metastases

Across tissue compartments, 1,026 primary-tumor cores, 293 lymph-node metastasis cores, and 37 distant-metastasis cores carried an evaluable TF H-score. In 247 patients with an assessable primary tumor and a lymph-node metastasis from the same resection, TF grade agreed moderately and showed no net shift between the two sites (Fig. 1D; Additional file 1: Table S5). The three-tier grade agreed in 61.1% (linearly weighted κ 0.51, 95% CI 0.41–0.59). Agreement was 72.9% at the positivity threshold (κ 0.46, 95% CI 0.34–0.57) and 83.4% at the TF-high threshold (κ 0.57, 95% CI 0.44–0.68), and H-scores correlated (Spearman ρ 0.66). TF-positive rates were 55.9% in primary tumors and 51.4% in lymph-node metastases (McNemar *p* = 0.22), TF-high rates 25.1% and 27.1% (*p* = 0.53), and the paired H-scores did not differ (median 20 and 20; Wilcoxon *p* = 0.91). Discordant grades ran in both directions (44 patients with a higher grade in the node, 52 with a higher grade in the primary tumor). Agreement was similar in treatment-naïve (*n* = 82; weighted κ 0.53) and pretreated patients (*n* = 154; weighted κ 0.52), and two cores from the same primary tumor agreed to a similar degree (14 of 20; weighted κ 0.56). Across all cores, median H-scores were 0 in primary tumors, 20 in lymph-node metastases and 35 in distant metastases. The primary tumors of patients with a nodal core also had a median H-score of 20, so the unpaired difference reflects which patients contributed metastasis cores. Only 10 patients had both a distant-metastasis core and an assessable primary tumor, too few for a formal comparison.

### TF expression after neoadjuvant therapy and tumor regression

**Fig. 2:**
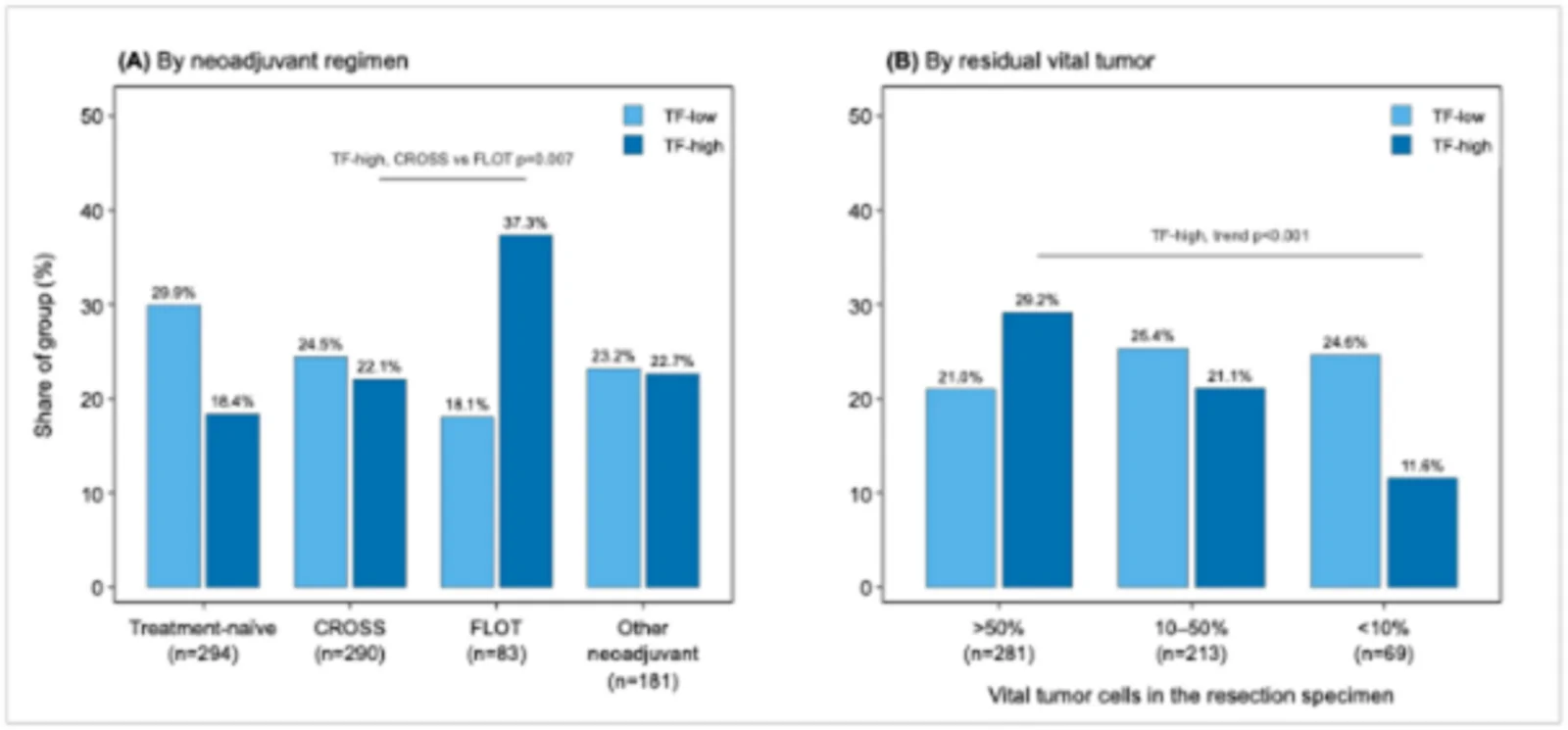
TF expression after neoadjuvant therapy. **A** Proportion of TF-low and TF-high tumors within each neoadjuvant treatment group (treatment-naïve, *n* = 294; CROSS, *n* = 290; FLOT, *n* = 83; other neoadjuvant, *n* = 181). TF positivity was similar in treatment-naïve tumors and in residual tumors after any neoadjuvant treatment (48.3% vs. 47.7%; Fisher exact *p* = 0.89; H-score *p* = 0.61), but TF-high tumors were more frequent after FLOT than after CROSS (37.3% vs. 22.1%; *p* = 0.007). **B** The same TF grades by histopathologic tumor regression, assessable only in pretreated tumors and expressed as the proportion of vital (residual) tumor cells in the resection specimen: more than 50% (*n* = 281), 10% to 50% (*n* = 213), and less than 10% (*n* = 69). The TF-high share falls as residual vital tumor decreases (29.2%, 21.1%, 11.6%; Cochran–Armitage trend *p* < 0.001), an association that persists after adjustment for ypT and ypN category (OR 0.64 per category, 95% CI 0.47–0.88; *p* = 0.007). TF-negative tumors make up the remainder of each group and are not plotted. All comparisons are cross-sectional on post-treatment residual tumor, not paired pre- and post-treatment samples. Patients with a complete pathologic response are not represented

Because all specimens were surgical resections, TF expression in treated patients reflects residual tumor after neoadjuvant therapy. TF positivity did not differ between treatment-naïve tumors and residual tumors after any neoadjuvant treatment (48.3% vs. 47.7%; *p* = 0.885), nor did the H-score distribution (Mann–Whitney U, *p* = 0.607). Treatment-naïve tumors that were locally advanced at resection (pT3–4 or pN+; *n* = 188) were TF-positive in 53.2% and TF-high in 23.9%, compared with 47.7% and 24.5% of pretreated residual tumors (*p* = 0.21 and *p* = 0.92). This group is an imperfect proxy for the patients who now receive neoadjuvant therapy. The distribution of the three TF grades differed modestly between treatment-naïve and pretreated tumors (*p* = 0.033): tumors treated with perioperative FLOT contained a higher proportion of TF-high cases than tumors treated with CROSS (37.3% vs. 22.1%; *p* = 0.007), and the overall grade distribution also separated FLOT from CROSS (*p* = 0.018) (Fig. 2A). The difference persisted after adjustment for ypT, ypN and regression grade (OR 2.19, 95% CI 1.20–4.00; *p* = 0.011) and was attenuated by further adjustment for year of operation (OR 1.95, 95% CI 1.01–3.76; *p* = 0.046). In patients operated in 2010 or later, TF-high rates were 21.8% after CROSS and 37.3% after FLOT (OR 2.14, 95% CI 1.24–3.70; adjusted for ypT, ypN and regression grade, OR 2.34, 95% CI 1.25–4.39). The complete-case models excluded 76 patients, 71 of them without a regression grade (Additional file 1: Table S6). FLOT-treated patients were operated later (median 2017 vs. 2014). Allocation was not randomized and the FLOT group was small (*n* = 83), so the finding is hypothesis-generating.

Histopathologic regression was assessable in 563 pretreated patients. TF-high expression concentrated in the tumors that had responded least: it was present in 29.2% of tumors with more than 50% residual vital tumor, 21.1% of those with 10% to 50%, and 11.6% of those with less than 10% (Cochran–Armitage trend *p* < 0.001; χ² *p* = 0.004 for TF-high versus other tumors), and median H-scores followed the same direction (15, 0 and 0; Kruskal–Wallis *p* = 0.008) (Fig. 2B). The gradient was carried by high-level expression: the TF-positive fraction at the > 10 threshold did not differ significantly across the three regression categories (50.2%, 46.5% and 36.2%; *p* = 0.11). In logistic regression adjusted for ypT and ypN category, each step towards less residual vital tumor remained associated with lower odds of TF-high expression (OR 0.64, 95% CI 0.47–0.88; *p* = 0.007), so the association is not explained by residual tumor stage. Regression was distributed similarly after CROSS and FLOT, with a non-significant excess of poorly regressed tumors after FLOT (more than 50% residual vital tumor in 54.5% vs. 40.3%; *p* = 0.10).

### Association with clinically actionable biomarkers

**Fig. 3:**
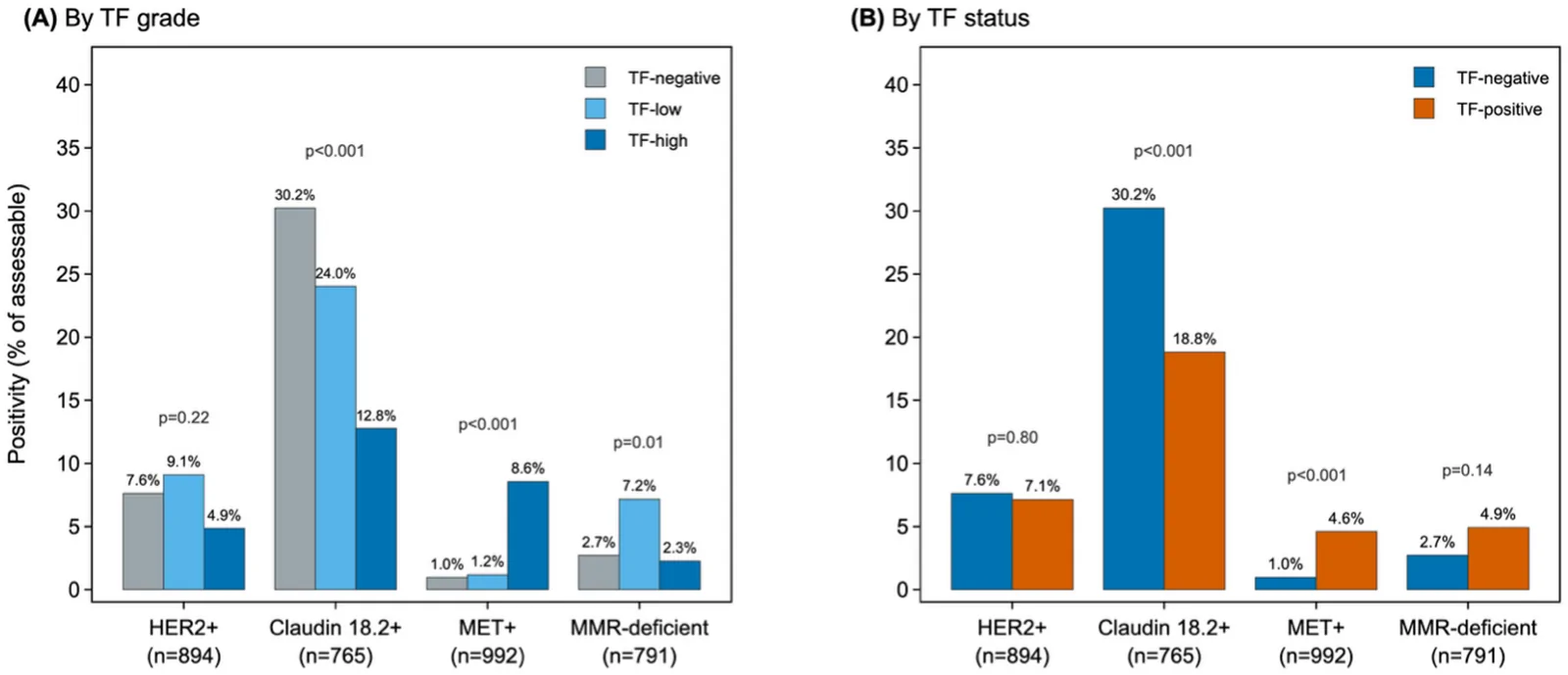
Association of TF expression with clinically actionable biomarkers. Positivity for HER2, Claudin 18.2, MET, and mismatch-repair deficiency, shown by three-tier TF grade (**A**) and by binary TF status (**B**). Bars give the percentage of marker-assessable tumors within each stratum; the marker-assessable denominator is shown below each biomarker (HER2, *n* = 894; Claudin 18.2, *n* = 765; MET, *n* = 992; MMR, *n* = 791). p-values: χ² across the three TF grades (**A**); Fisher exact for TF-positive versus TF-negative (**B**)

To test whether TF marks a molecularly distinct subgroup, TF grade and TF status were compared with established and emerging therapeutic biomarkers (Fig. 3; Table 2). TF expression was independent of HER2 status: HER2 positivity was found in 7.1% of TF-positive and 7.6% of TF-negative tumors (*p* = 0.80), with no trend across TF grades (*p* = 0.22). CLDN18.2 positivity, by contrast, fell as TF expression rose, from 30.2% in TF-negative to 24.0% in TF-low and 12.8% in TF-high tumors (*p* < 0.001), giving an inverse association on the binary comparison as well (18.8% of TF-positive vs. 30.2% of TF-negative; *p* < 0.001). MET overexpression showed the opposite pattern and concentrated in the TF-high group, rising from 1.0% in TF-negative and 1.2% in TF-low tumors to 8.6% in TF-high tumors (*p* < 0.001; 4.6% of TF-positive vs. 1.0% of TF-negative, *p* < 0.001). Mismatch-repair deficiency (MLH1 loss) also differed across TF grades (*p* = 0.01), but the relationship was non-monotonic: prevalence was highest in TF-low tumors (7.2%) and comparable in TF-negative (2.7%) and TF-high (2.3%) tumors. On the binary comparison MMR deficiency did not differ by TF status (4.9% of TF-positive vs. 2.7% of TF-negative; *p* = 0.14). This non-linear pattern rests on a small number of MMR-deficient cases and should be interpreted with caution.

**Table 2:** Association of clinically actionable molecular biomarkers with three-tier TF expression grade (1006 EAC/EGJ patients with an assessable primary-tumor TF H-score)

Biomarker	Overall (*n* = 1006)	TF-negative (*n* = 520)	TF-low (*n* = 261)	TF-high (*n* = 225)	*p*
**HER2+**	66 (7.4%)	34 (7.6%)	22 (9.1%)	10 (4.9%)	0.22
Not assessable, n (%)	112 (11.1%)	74 (14.2%)	19 (7.3%)	19 (8.4%)	
**Claudin 18.2+**	187 (24.4%)	114 (30.2%)	50 (24.0%)	23 (12.8%)	< 0.001
Not assessable, n (%)	241 (24.0%)	143 (27.5%)	53 (20.3%)	45 (20.0%)	
**MET+**	27 (2.7%)	5 (1.0%)	3 (1.2%)	19 (8.6%)	< 0.001
Not assessable, n (%)	14 (1.4%)	7 (1.3%)	4 (1.5%)	3 (1.3%)	
**MMR-deficient**	30 (3.8%)	11 (2.7%)	15 (7.2%)	4 (2.3%)	0.01
Not assessable, n (%)	215 (21.4%)	115 (22.1%)	52 (19.9%)	48 (21.3%)	

Cells are positive n (% of marker-assessable within stratum); denominators vary by marker and are given by the not-assessable line beneath eachmarker. Marker positivity defined as in Methods. P-values: χ² across the three TF grades

### TF expression and overall survival

**Fig. 4:**
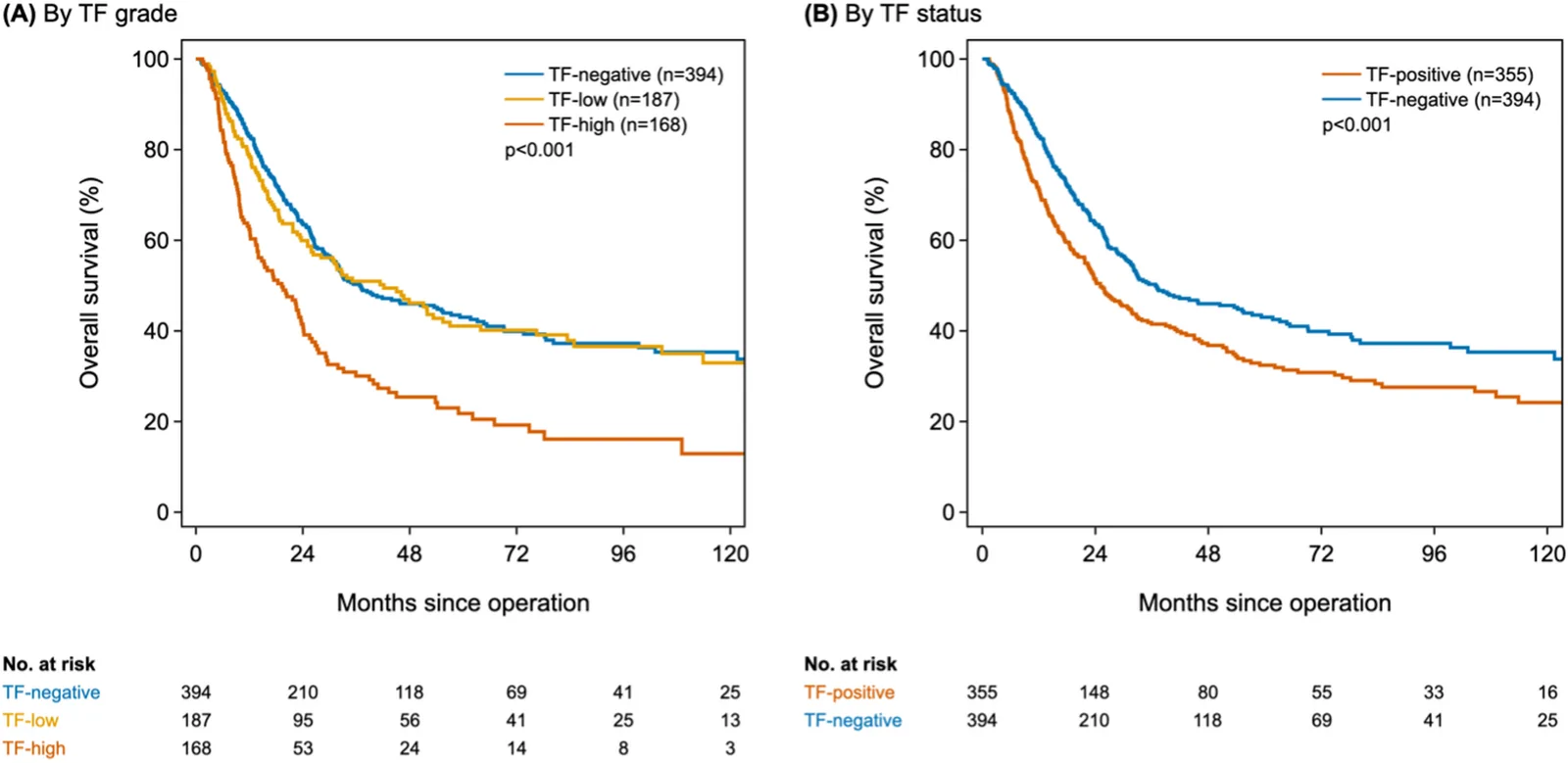
Overall survival by TF expression (*n* = 749; 428 deaths). Kaplan–Meier estimates by three-tier TF grade (**A**; TF-negative *n* = 394, TF-low *n* = 187, TF-high *n* = 168) and by binary TF status (**B**; TF-positive *n* = 355, TF-negative *n* = 394), with numbers at risk below each panel. p-values by log-rank test. Median overall survival was 36.8, 42.2, and 19.3 months for TF-negative, TF-low, and TF-high tumors, respectively

Overall survival was analyzed in 749 patients with follow-up and assessable TF expression; 428 deaths (57%) occurred during follow-up. The 257 patients without follow-up were not in the institutional follow-up database (*n* = 157), had no recorded follow-up (*n* = 99) or had a conflicting entry (*n* = 1) (Additional file 1: Figure S3). Their TF grade, age and sex matched those of the analyzed patients (TF-high 22.2% and 22.4%; *p* = 0.46). They more often had pN2 disease (21.4% vs. 14.8%) and were operated later (median 2017 vs. 2013), including 90 of the 91 patients operated in 2020 (Additional file 1: Table S2). TF-high tumors had a markedly worse prognosis than TF-low or TF-negative tumors (log-rank *p* < 0.001) (Fig. 4A). Median overall survival was 19.3 months for TF-high tumors, versus 36.8 months for TF-negative and 42.2 months for TF-low tumors; corresponding 5-year survival rates were 22%, 43%, and 41%. TF-low and TF-negative tumors were thus prognostically similar, and the survival detriment was confined to the TF-high group. On the binary classification, TF-positive tumors also had shorter overall survival than TF-negative tumors (log-rank *p* < 0.001; Fig. 4B), with a univariable hazard ratio of 1.39 (95% CI 1.15–1.68; *p* < 0.001). Because the > 10 threshold merges the prognostically neutral TF-low group with TF-high tumors, it understates the effect seen at higher expression: univariable hazard ratios were 1.49 at H-score > 50, 1.90 at > 100, and 1.89 at > 150, each highly significant (*p* < 0.001), with corresponding stage-adjusted hazard ratios of 1.37, 1.68 and 1.53. Across the full grid of thresholds from > 10 to > 250, the adjusted hazard ratio for TF-high tumors remained significantly raised at every cut-off tested (all *p* ≤ 0.031), so the survival finding does not depend on where the boundary is drawn; the log-rank statistic was maximal at the adopted threshold of 100 (χ²=35.2), which, because the threshold had been chosen without reference to outcome, corroborates rather than defines it (Additional file 1: Figure S2, Table S1).

Analyzed as a continuous variable, the TF H-score was associated with shorter overall survival after adjustment (HR 1.11 per 50 points, 95% CI 1.06–1.18; *p* < 0.001). In a restricted cubic spline, the hazard did not rise up to an H-score of about 70, increased between about 70 and 200 and leveled off above that, where data were sparse. Departure from linearity was not significant (*p* = 0.18 with four knots, *p* = 0.69 with three). The three-tier grade fitted at least as well as the linear term (AIC 4836 vs. 4840; Additional file 1: Figure S4).

**Fig. 5:**
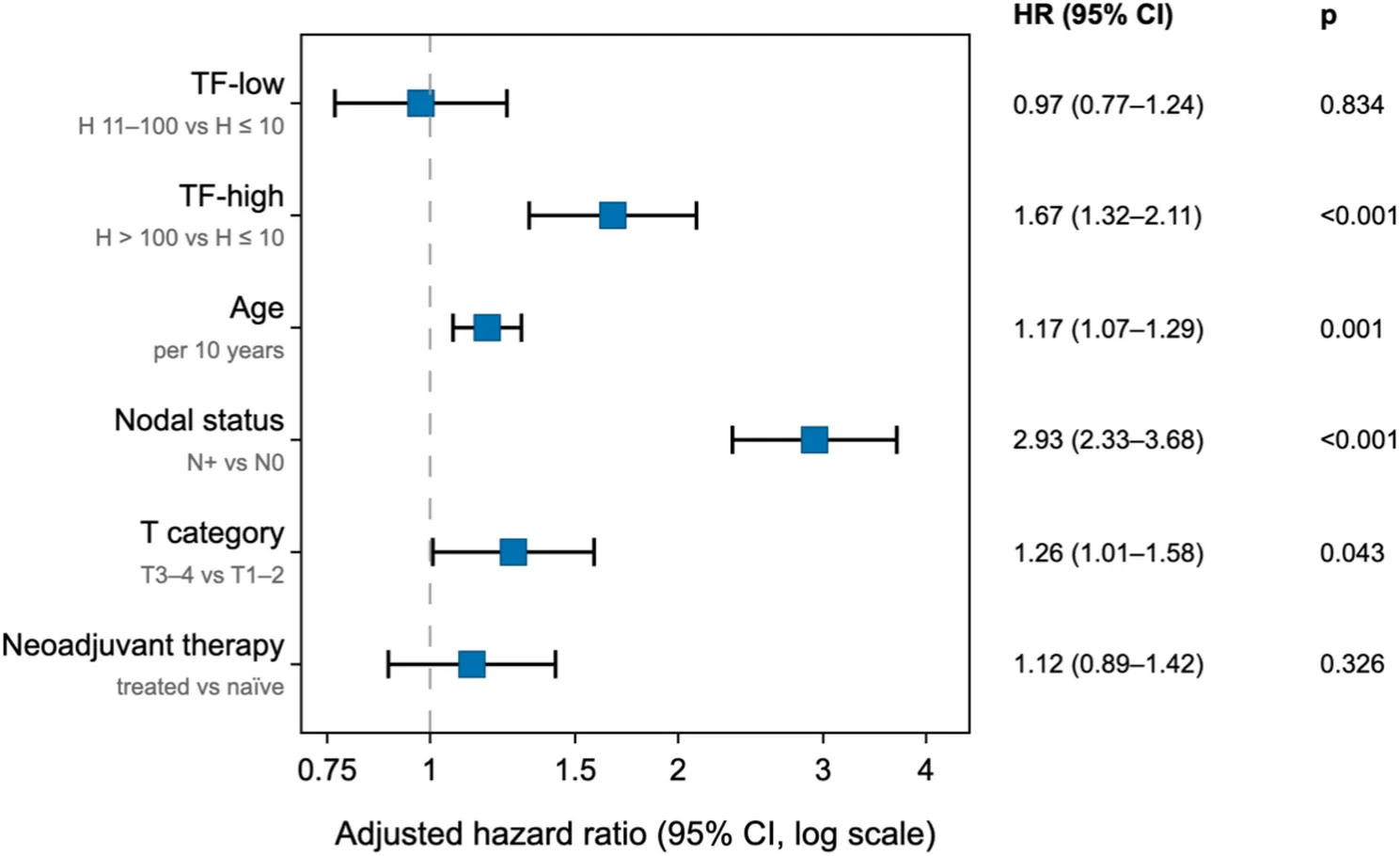
Multivariable Cox regression for overall survival (*n* = 730; 421 deaths). Adjusted hazard ratios (squares) with 95% confidence intervals (horizontal lines) for the three-tier TF grade and clinicopathologic covariates; the dashed line marks a hazard ratio of 1. Reference categories are TF-negative (H-score ≤ 10), N0, T1–2, and treatment-naïve. The adverse hazard is confined to TF-high tumors (HR 1.67, 95% CI 1.32–2.11; *p* < 0.001); TF-low tumors do not differ from TF-negative tumors (HR 0.97, 95% CI 0.77–1.24; *p* = 0.83)

In multivariable Cox regression including the three-tier TF grade, age, pT category, nodal status, and neoadjuvant therapy (730 patients, 421 deaths), the adverse effect of TF was confined to the highest expression tier: TF-high tumors carried an independently increased risk of death compared with TF-negative tumors (HR 1.67, 95% CI 1.32–2.11; *p* < 0.001), whereas TF-low tumors were indistinguishable from TF-negative tumors (HR 0.97, 95% CI 0.77–1.24; *p* = 0.83) (Fig. 5). Nodal positivity (HR 2.93, 95% CI 2.33–3.68; *p* < 0.001), older age (HR 1.17 per 10 years, 95% CI 1.07–1.29; *p* = 0.001), and advanced T category (HR 1.26, 95% CI 1.01–1.58; *p* = 0.043) were also independent predictors, whereas neoadjuvant therapy was not (HR 1.12, 95% CI 0.89–1.42; *p* = 0.33). Entered instead as a binary variable, TF positivity likewise remained independently adverse, but with a smaller effect estimate (HR 1.25, 95% CI 1.03–1.51; *p* = 0.026), reflecting the dilution of the TF-high hazard by the prognostically neutral TF-low group. The proportional-hazards assumption was satisfied for all covariates (Schoenfeld residuals, all *p* ≥ 0.21).

In sensitivity analyses, the TF-high hazard ratio stayed between 1.62 and 1.79 when year of operation, treatment era (as covariate or stratum), resection margin, tumor grade, lymphatic or venous invasion or sex was added, after exclusion of the 26 patients with documented pM1 disease, and with the mean instead of the highest-scoring core (Additional file 1: Table S3). With margin, lymphovascular invasion, sex and era combined (*n* = 484), it was 1.76 (95% CI 1.31–2.37); with grade also added (*n* = 275), it was 2.12 (95% CI 1.41–3.19). The primary model refitted in the same patients gave 1.69 and 2.24, so these shifts reflect which patients had complete pathology data, and the added covariates changed the estimate little. The same holds for TF-low. Its hazard ratio was 0.96 to 0.99 in all models on the 730 patients and 1.26 to 1.78 in the complete-case subsets with margin or grade, where the primary model without these covariates already gave 1.22 to 1.68.

Hazard ratios for TF-high versus all other tumors exceeded 1 in every subgroup except the earliest era, without evidence of effect modification (interaction *p* = 0.14 to 0.91; Additional file 1: Figure S5). They were 1.66 (95% CI 0.93–2.95) in pT3–4N0 and 2.14 (95% CI 1.16–3.91) in pT1–2 N+ tumors, 1.77 (95% CI 1.12–2.78) in treatment-naïve and 1.64 (95% CI 1.28–2.11) in pretreated patients, and 1.34 (95% CI 0.95–1.88) after CROSS and 2.53 (95% CI 1.41–4.54) after FLOT. Several subgroups were small: the 1996–2005 era had 11 TF-high patients (HR 1.02, 95% CI 0.46–2.28) and the pT1–2N0 group 16. In the 419 of 517 pretreated patients with a regression grade, TF-high remained prognostic with regression grade in the model (HR 1.49, 95% CI 1.10–2.01), whereas regression grade itself was not (HR 0.93 per category, 95% CI 0.77–1.11; Additional file 1: Table S6).

Taken together, these data show TF expression to be common in EAC and to mark tumors with advanced stage and a distinct biomarker profile, with moderate agreement between primary tumors and their lymph-node metastases; at high expression levels it is independently associated with shortened overall survival.

## Discussion

In the present study, we comprehensively analyzed Tissue Factor (TF/CD142) expression in a large cohort of more than 1,000 esophageal adenocarcinomas (EAC) and adenocarcinomas of the esophagogastric junction (EGJ), representing, to the best of our knowledge, the largest study specifically investigating TF expression in this disease entity to date. Several clinically relevant findings emerged from this analysis. First, TF expression was common, with approximately half of all tumors demonstrating detectable expression and reaching high levels in roughly one-quarter. Second, TF positivity was associated with advanced local tumor growth and nodal dissemination, but the survival detriment was confined to tumors with high-level expression. Third, high TF expression remained an independent adverse prognostic factor after adjustment for established clinicopathological variables, whereas TF-low tumors were prognostically indistinguishable from TF-negative tumors. Finally, TF expression demonstrated distinct relationships with clinically actionable biomarkers, highlighting its potential biological and therapeutic relevance in EAC. TF grade agreed moderately between primary tumors and their synchronous lymph-node metastases, without a net shift. These are prognostic findings. Whether TF-high tumors respond to TF-directed ADCs is a separate question that this cohort cannot answer.

A TF-positive rate near 48% places EAC in the middle of the range reported across gastrointestinal cancers, where immunohistochemical TF positivity runs from roughly 27% in a European gastric series [32] to 76–86% in colorectal and pancreatic cancer [25, 33]. It is also close to the 63% reported for the esophageal cohort of the largest cross-tumor TF survey, which used an H-score and a comparable “any unequivocal staining” cutoff — but that cohort comprised only 62 cases of unspecified histology [25]. The only prior study dedicated to esophageal TF examined 67 squamous cell carcinomas and used TF to predict chemotherapy resistance [24]. Neither speaks to Western, Barrett-associated adenocarcinoma. Our cohort therefore fills a specific gap, and the prevalence it establishes (nearly one in two tumors, one in four at high level) defines the size of the population that TF-directed therapy could in principle reach.

The prognostic effect was dose-dependent and driven by the TF-high group. TF-low tumors were indistinguishable from TF-negative tumors, whereas TF-high tumors had a median overall survival of 19.3 months versus roughly 37–42 months for the other two groups. Correspondingly, the univariable hazard ratio rose from 1.39 at the > 10 positivity threshold to about 1.90 at an H-score > 100. After adjustment for age, T and N category and neoadjuvant therapy, the excess risk was confined to TF-high tumors (HR 1.67), while TF-low tumors were indistinguishable from TF-negative (HR 0.97). This direction and independence are consistent with prior work in gastrointestinal adenocarcinoma, where high tumoral TF has been an adverse prognostic factor in intestinal-type gastric cancer (the phenotype EAC/EGJ shares [34]) in colorectal cancer [33], and in pancreatic cancer [35]. The continuous analysis gave the same picture: the hazard did not rise over the low H-score range and increased at higher expression, so the association does not depend on the categorization. In pretreated patients, TF-high remained prognostic after adjustment for regression grade, so it is not only a marker of poor response. Because the binary > 10 threshold merges prognostically neutral TF-low tumors with TF-high tumors, it understates the effect, a point with practical bearing: if a TF cutoff is ever used to select therapy, the biology and the survival data both argue for setting it at high expression rather than at any detectable staining. No published threshold for high TF expression exists against which our boundary could be benchmarked. Prior immunohistochemical studies of TF have dichotomized at intense staining in more than 10% of tumor cells [32, 34], and the largest cross-tumor survey, although it used the same 0 to 300 H-score, likewise classified tumors by the proportion of stained cells rather than by an H-score value [25]. Trials of TF-directed antibody–drug conjugates offer no anchor either: InnovaTV 201 found no relationship between TF expression and response [22], and the MRG004A study required only documented TF staining for enrolment [23]. Our > 10 positivity threshold is thus consistent with the prevailing “any unequivocal staining” convention, whereas the > 100 boundary is, to our knowledge, the first quantitative high-expression threshold proposed for TF. It was derived without reference to outcome, it coincided with the threshold that maximized the log-rank statistic in this cohort, and the adverse hazard was present at every alternative threshold we tested, but it is a single-cohort proposal and would need to be reproduced before it could anchor an assay.

TF-positive tumors were significantly associated with advanced pT and pN categories, consistent with the established role of TF in tumor invasion, angiogenesis, coagulation-mediated tumor progression, and metastatic dissemination. Experimental studies have demonstrated that TF signaling extends far beyond activation of the coagulation cascade and contributes to multiple hallmarks of cancer through activation of protease-activated receptor pathways, modulation of the tumor microenvironment, and promotion of tumor cell migration and invasion. In our cohort, the unpaired comparison of all cores suggested higher H-scores in metastases. Within patients, there was no net shift between primary tumor and synchronous lymph-node metastasis, and agreement was moderate (κ 0.46 to 0.57). Node-positive tumors expressed more TF, and the primary tumors of these patients already had the same median H-score as their nodal metastases. The data therefore give no evidence that TF rises during nodal spread. Two cores from the same primary tumor agreed to a similar degree, so much of the discordance probably reflects sampling of a heterogeneous tumor. It still matters for testing: discordance affected 27% of patients at the positivity threshold and 17% at the TF-high threshold, so a result from one site predicts the other only moderately. Our nodal metastases were synchronous, and the data do not address metachronous recurrences, the setting in which a TF-directed ADC would be used. Distant metastases were too few (10 paired patients) for any conclusion.

Notably, TF expression was independent of HER2 status, so TF-directed therapies would not merely target the same patient population already eligible for HER2-directed treatment, but rather provide access to an additional and partially independent therapeutic subgroup. Similarly, CLDN18.2 positivity fell as TF rose, from 30% in TF-negative to 13% in TF-high tumors, which may indicate that TF-directed therapies could potentially address patients lacking CLDN18.2 expression and therefore not eligible for CLDN18.2-targeted treatment strategies. The association with MET ran in the opposite direction. MET overexpression was uncommon overall but concentrated almost entirely in TF-high tumors (about 9% versus 1% elsewhere). MET overexpression and amplification mark an aggressive, poor-prognosis subtype of gastroesophageal adenocarcinoma [30], and the MET-directed ADC telisotuzumab vedotin has accelerated approval in the United States for previously treated non-squamous non-small-cell lung cancer with high MET protein overexpression [36], and its topoisomerase-1 successor telisotuzumab adizutecan produced objective responses in roughly a quarter of heavily pretreated gastric/EGJ patients [37]. A TF-high, MET-enriched compartment thus identifies tumors that might be susceptible to either target, and is a natural subgroup for future co-expression and combination studies. Mismatch-repair deficiency, by contrast, showed no coherent relationship with TF (most frequent in TF-low tumors and similar in TF-negative and TF-high tumors) and with only 30 deficient cases we read this as a null result rather than a biological pattern.

The relationship between TF expression and neoadjuvant treatment needs careful reading. TF expression remains prevalent in the residual tumor from which a recurrence would arise, and it remains assessable in the resection specimen. The comparison with treatment-naïve tumors involves different patients, so it does not show that TF persists within an individual tumor. It also excludes patients with a complete pathologic response, who made up 10% to 17% of patients after CROSS or FLOT in ESOPEC [4]. TF-high expression was most frequent in poorly regressed tumors, in line with the only prior esophageal TF study, in squamous cell carcinoma, in which TF in pretreatment biopsies predicted chemoresistance [24]. Complete responders were therefore probably enriched for TF-low or TF-negative tumors, so the TF-high share in residual tumor may overstate the pretreatment share. The bias is limited: even if every complete responder had been TF-negative, the pretreatment TF-high share would have been about 20% to 22% instead of 24.5%. Treatment-naïve tumors that were locally advanced at resection had similar TF rates, but these patients differ from those now selected for neoadjuvant therapy in era, fitness and clinical stage, so the comparison neither confirms nor refutes the pretreatment prevalence. Paired diagnostic biopsies and resection specimens would be needed to measure it and any change in TF status under therapy.

TF-high tumors were more frequent after FLOT than after CROSS. Measured differences in residual stage and regression grade did not explain this, and the difference remained in patients operated in 2010 or later. Confounding by tumor location, which guides the choice of regimen and was not available, remains likely. Allocation was not randomized and FLOT-treated patients were few, so the finding is a hypothesis for paired, ideally prospective, studies. It matters because FLOT-based regimens are now standard for resectable esophageal and EGJ adenocarcinoma [4, 5].

Several limitations apply. The retrospective design precludes conclusions about causality, and the treatment-related analyses compare residual tumor of different patients rather than matched pre- and post-treatment samples. Most patients were represented by a single 1.2-mm core, which samples a small part of a tumor whose TF expression can be heterogeneous. In the 20 patients with two primary cores, the grade agreed in 14. Using the mean instead of the higher score changed the grade of 2 patients (TF-high 223 vs. 225) and left the survival estimate unchanged. The highest-scoring-core rule therefore did not materially raise the prevalence in this cohort, but a single core can misclassify a heterogeneous tumor in either direction, and prevalence on whole sections or diagnostic biopsies, the material used for treatment decisions, may differ. Follow-up was missing for a quarter of patients, mostly those operated most recently; their TF grade distribution matched that of the analyzed patients. Only overall survival was available, so disease-specific survival and competing risks could not be analyzed. The cohort spans 1996 to 2020. Adjustment for and stratification by treatment era did not change the result, but residual confounding by changes in staging, surgery and treatment of recurrence cannot be excluded. The earliest era contributed few TF-high patients, and its hazard ratio was imprecise (1.02). Loss of antigenicity in older blocks would lower TF expression in early years regardless of treatment; the absence of a time trend in treatment-naïve tumors argues against this but cannot exclude it. Tumor location and pretreatment clinical stage were not available, and resection margin, grade and lymphovascular invasion were incompletely recorded.

Most important, no prospective data yet link TF expression in EAC to benefit from TF-directed therapy, so the predictive value of the assay (as opposed to its prognostic value, which we demonstrate) remains to be established. The MET and mismatch-repair subgroups were small. The TF-high threshold, although fixed on the marker distribution rather than on outcome and robust across the whole range of alternatives tested, was defined within this cohort and requires validation in an independent series, ideally alongside a standardized assay, before it could serve as a clinical cut-point. Set against these limitations are the study’s size, its histologically defined adenocarcinoma cohort, the paired comparison of primary tumors and nodal metastases, an integrated panel of contemporary therapeutic biomarkers, and a standardized H-score readout.

In conclusion, TF is expressed in nearly half of EAC and EGJ adenocarcinomas and is associated with advanced tumor stage, and, at high expression, independently shorter overall survival, and defines a molecular compartment that is largely separate from HER2 and CLDN18.2 and enriched for MET. TF expression remains prevalent in residual tumor after neoadjuvant therapy. These data identify TF-high as a prognostic subgroup and a candidate biomarker for prospective trials of TF-directed ADCs in esophageal and EGJ adenocarcinoma, in which the H-score threshold should be validated and its predictive value tested.

## Supplementary Information

Below is the link to the electronic supplementary material.

Supplementary Material 1

## Data Availability

The data supporting the findings of this study are available from the corresponding author upon reasonable request.

## Abbreviations

ADC: antibody–drug conjugate
AIC: Akaike’s information criterion
CI: confidence interval
CLDN18.2: Claudin 18.2
CROSS: Chemoradiotherapy for Oesophageal Cancer followed by Surgery Study
dMMR: mismatch repair-deficient
EAC: esophageal adenocarcinoma
EGJ: esophagogastric junction
FISH: fluorescence in situ hybridization
FLOT: fluorouracil, leucovorin, oxaliplatin and docetaxel
HER2: human epidermal growth factor receptor 2
HR: hazard ratio
IHC: immunohistochemistry
IQR: interquartile range
MMR: mismatch repair
MSI: microsatellite instability
OR: odds ratio
OS: overall survival
PD-L1: programmed death-ligand 1
pMMR: mismatch repair-proficient
TF: Tissue Factor
TMA: tissue microarray
