# Supplementary material for: High tissue factor (CD142) expression identifies a poor-prognosis subgroup of esophageal and esophagogastric junction adenocarcinoma
Source: BMC Cancer. 2026 Nov 10;26:1379. doi: 10.1186/s12885-026-17336-w (PMC13749868; doi:10.1186/s12885-026-17336-w)
Supplement: Supplementary file 1 [file 12885_2026_17336_MOESM1_ESM.docx]

**Additional file 1**

High Tissue Factor (CD142) Expression Identifies a Poor-Prognosis Subgroup of Esophageal and Esophagogastric Junction Adenocarcinoma

Supplementary Figures S1–S5 and Supplementary Tables S1–S6, numbered in order of first citation in the manuscript.

**Figure S1. Sensitivity of the TF-positive fraction to the H-score cut-off.** Percentage of assessable primary tumors (n=1,006) classified as TF-positive at H-score cut-offs of >0, >10 (adopted), >50, and >100. The fraction is near-identical at >0 and >10, supporting the adopted >10 threshold, and reaches 22.4% at >100 (the TF-high band).

**Figure S2. Sensitivity of the TF-high survival signal to the H-score cut-off (749 patients with follow-up, 428 deaths).** (A) Hazard ratio for TF-high tumors versus all remaining patients across thresholds from an H-score >10 to >250, unadjusted (dotted line) and adjusted for age, pT category, nodal status and neoadjuvant therapy (solid line, shaded 95% confidence interval). The adjusted hazard is raised at every threshold tested (all p≤0.031), so the survival conclusion is independent of where the TF-low/TF-high boundary is drawn. (B) Log-rank χ² across the same grid, maximal at an H-score >100 — the adopted cut-off, which had been fixed on the median H-score of the TF-positive tumors rather than on outcome. Dashed vertical line, the adopted threshold of 100.

**Figure S3. Flow of patients through the study.** Of 1,120 patients on the tissue microarray, 1,006 had an assessable primary-tumor TF H-score (analysis cohort), 749 had follow-up (428 deaths) and 730 had complete covariates for the multivariable Cox model (421 deaths). Reasons for exclusion are given at each step. Missing-covariate counts overlap because some patients lacked more than one. Patients without follow-up are compared with the analyzed patients in Table S2.

**Figure S4. TF H-score as a continuous variable (730 patients, 421 deaths).** Hazard ratio for overall survival relative to an H-score of 0 from a restricted cubic spline with four knots (H-scores 20, 60, 150, 300, Harrell’s default percentiles of the H-score among TF-positive tumors), adjusted for age, pT category, nodal status and neoadjuvant therapy; shading, 95% confidence interval. Dotted lines mark the positivity threshold (H-score 10) and the TF-low/TF-high boundary (H-score 100). Few tumors scored above 250, so the curve is imprecise there. Overall association p<0.001; non-linearity p=0.18 (p=0.69 with three knots). The linear term gives HR 1.11 per 50 points (95% CI 1.06–1.18).

**Figure S5. TF-high and overall survival by subgroup (730 patients, 421 deaths).** Hazard ratio for TF-high versus all other tumors (squares, 95% confidence intervals) within each subgroup, adjusted for the primary-model covariates that vary within it (age, pT category, nodal status, neoadjuvant therapy). Labels give TF-high patients/all patients. Interaction p-values: likelihood-ratio test for TF-high × subgroup; the regimen interaction is tested among pretreated patients. Treatment era is the year of operation. The 1996–2005 and pT1–2N0 subgroups contain few TF-high patients.

**Table S1. Sensitivity of the TF-high definition to the H-score cut-off.** The adopted boundary (H-score > 100) is the median H-score of the 486 TF-positive tumors (IQR 40–180) and was therefore fixed on the marker distribution alone, without reference to survival. TF-high n (%) is of the 1,006 assessable primary tumors; survival statistics are from the 749 patients with follow-up (428 deaths), comparing TF-high with all remaining patients at each threshold. Adjusted models carry age (per 10 years), pT (T3–4 vs T1–2), nodal status (N+ vs N0) and neoadjuvant therapy (treated vs naïve) and use the same 730 complete cases throughout. The adverse hazard persists at every threshold tested, and the log-rank statistic is maximal at the adopted cut-off (χ² = 35.2 at H > 100); because the cut-off was not selected on outcome, this coincidence corroborates rather than defines it. All p-values are two-sided and unadjusted for multiple comparisons.

| **H-score cut-off** | **TF-high, n (%)** | **Median OS, TF-high, mo** | **Median OS, rest, mo** | **Log-rank χ²** | **Unadjusted HR (95% CI)** | **Univ. p** | **Adjusted HR (95% CI)** | **Multiv. p** |
| --- | --- | --- | --- | --- | --- | --- | --- | --- |
| > 10 | 486 (48.3%) | 25.1 | 36.8 | 11.7 | 1.39 (1.15–1.68) | <0.001 | 1.25 (1.03–1.51) | 0.03 |
| > 25 | 420 (41.7%) | 24.2 | 37.1 | 12.0 | 1.40 (1.16–1.70) | <0.001 | 1.29 (1.06–1.57) | 0.01 |
| > 50 | 323 (32.1%) | 23.0 | 36.8 | 16.0 | 1.49 (1.23–1.82) | <0.001 | 1.37 (1.12–1.68) | 0.002 |
| > 75 | 282 (28.0%) | 21.7 | 37.2 | 27.1 | 1.71 (1.39–2.09) | <0.001 | 1.57 (1.28–1.94) | <0.001 |
| > 100 (adopted) | 225 (22.4%) | 19.3 | 37.2 | 35.2 | 1.90 (1.53–2.35) | <0.001 | 1.68 (1.35–2.09) | <0.001 |
| > 125 | 183 (18.2%) | 17.5 | 34.1 | 25.3 | 1.80 (1.43–2.28) | <0.001 | 1.52 (1.20–1.93) | <0.001 |
| > 150 | 146 (14.5%) | 16.0 | 33.3 | 24.6 | 1.89 (1.47–2.45) | <0.001 | 1.53 (1.18–1.99) | 0.001 |
| > 175 | 122 (12.1%) | 15.3 | 33.3 | 30.9 | 2.13 (1.62–2.79) | <0.001 | 1.69 (1.28–2.23) | <0.001 |
| > 200 | 96 (9.5%) | 17.5 | 32.8 | 21.9 | 2.01 (1.49–2.70) | <0.001 | 1.59 (1.18–2.15) | 0.002 |

**Table S2. Patients with and without follow-up.** Patients of the analysis cohort (n=1,006) with follow-up (included in the survival analysis) and without follow-up (excluded). Percentages are of the column total.

| **Characteristic** | **Included (n=749)** | **Excluded (n=257)** | **p** |
| --- | --- | --- | --- |
| **TF grade, n (%)** |  |  | 0.46 |
| TF-negative | 394 (52.6%) | 126 (49.0%) |  |
| TF-low | 187 (25.0%) | 74 (28.8%) |  |
| TF-high | 168 (22.4%) | 57 (22.2%) |  |
| **Age at operation, median [IQR], y** | 64 [56–71] | 65 [56–71] | 0.38 |
| **Sex, n (%)** |  |  | 0.84 |
| Male | 660 (88.1%) | 224 (87.2%) |  |
| Female | 88 (11.7%) | 32 (12.5%) |  |
| Unknown | 1 (0.1%) | 1 (0.4%) |  |
| **pT category, n (%)** |  |  | 0.05 |
| T1 | 135 (18.0%) | 43 (16.7%) |  |
| T2 | 128 (17.1%) | 62 (24.1%) |  |
| T3 | 458 (61.1%) | 134 (52.1%) |  |
| T4 | 23 (3.1%) | 7 (2.7%) |  |
| Not assessable | 5 (0.7%) | 11 (4.3%) |  |
| **pN category, n (%)** |  |  | 0.04 |
| N0 | 293 (39.1%) | 81 (31.5%) |  |
| N1 | 216 (28.8%) | 70 (27.2%) |  |
| N2 | 111 (14.8%) | 55 (21.4%) |  |
| N3 | 116 (15.5%) | 38 (14.8%) |  |
| Not assessable | 13 (1.7%) | 13 (5.1%) |  |
| **Year of operation, median [IQR]** | 2013 [2009–2016] | 2017 [2010–2020] | <0.001 |
| **Treatment era, n (%)** |  |  | <0.001 |
| 1996–2005 | 67 (8.9%) | 37 (14.4%) |  |
| 2006–2012 | 260 (34.7%) | 52 (20.2%) |  |
| 2013–2020 | 422 (56.3%) | 168 (65.4%) |  |
| **Resection margin, n (%)** |  |  | 0.08 |
| R0 | 540 (72.1%) | 177 (68.9%) |  |
| R1–2 | 37 (4.9%) | 21 (8.2%) |  |
| Not recorded | 172 (23.0%) | 59 (23.0%) |  |
| **Neoadjuvant therapy, n (%)** |  |  | n/a |
| Treatment-naïve | 232 (31.0%) | 62 (24.1%) |  |
| CROSS | 282 (37.7%) | 8 (3.1%) |  |
| FLOT | 83 (11.1%) | 0 (0.0%) |  |
| Other neoadjuvant | 152 (20.3%) | 29 (11.3%) |  |
| Unknown | 0 (0.0%) | 158 (61.5%) |  |

P-values: χ² (categorical) or Mann–Whitney U (continuous). Unknown, not-assessable and not-recorded categories are shown but excluded from the tests; not-assessable pT and pN were more frequent among excluded patients. Neoadjuvant treatment is recorded in the follow-up database and is missing by design for most excluded patients, so it is shown but not tested (n/a).

**Table S3. Sensitivity analyses of the multivariable Cox model for overall survival.** Hazard ratios for TF-high and TF-low versus TF-negative tumors. The primary model includes age (per 10 years), pT category (T3–4 vs T1–2), nodal status (N+ vs N0) and neoadjuvant therapy (treated vs naïve); each further row adds the named terms or changes the population.

| **Model** | **Patients (deaths)** | **TF-high HR (95% CI)** | **p** | **TF-low HR (95% CI)** | **p** | **Same patients, primary model: TF-high / TF-low HR** |
| --- | --- | --- | --- | --- | --- | --- |
| Primary model | 730 (421) | 1.67 (1.32–2.11) | <0.001 | 0.97 (0.77–1.24) | 0.83 |  |
| + year of operation (per 5 years) | 730 (421) | 1.67 (1.32–2.10) | <0.001 | 0.96 (0.75–1.22) | 0.74 |  |
| + treatment era (3 periods) | 730 (421) | 1.66 (1.31–2.10) | <0.001 | 0.97 (0.76–1.23) | 0.79 |  |
| Stratified by treatment era | 730 (421) | 1.66 (1.31–2.10) | <0.001 | 0.98 (0.77–1.24) | 0.84 |  |
| + resection margin (R1–2 vs R0) | 569 (316) | 1.74 (1.33–2.29) | <0.001 | 1.26 (0.96–1.65) | 0.10 | 1.69 / 1.22 |
| + tumor grade (G3–4 vs G1–2) | 418 (249) | 1.79 (1.31–2.45) | <0.001 | 1.31 (0.96–1.78) | 0.08 | 1.89 / 1.30 |
| + lymphatic invasion (L1) | 560 (316) | 1.76 (1.35–2.30) | <0.001 | 1.03 (0.78–1.36) | 0.81 | 1.73 / 1.02 |
| + venous invasion (V1) | 546 (301) | 1.77 (1.35–2.32) | <0.001 | 1.05 (0.79–1.40) | 0.73 | 1.77 / 1.03 |
| + sex | 730 (421) | 1.62 (1.28–2.05) | <0.001 | 0.99 (0.77–1.25) | 0.90 |  |
| + R, L, V, sex and era | 484 (264) | 1.76 (1.31–2.37) | <0.001 | 1.35 (0.99–1.83) | 0.06 | 1.69 / 1.26 |
| + R, L, V, sex, era and grade | 275 (160) | 2.12 (1.41–3.19) | <0.001 | 1.78 (1.21–2.62) | 0.003 | 2.24 / 1.68 |
| Excluding pM1 at resection | 704 (400) | 1.65 (1.30–2.10) | <0.001 | 0.97 (0.76–1.24) | 0.79 |  |
| Accession-key survival matches only | 728 (420) | 1.67 (1.32–2.11) | <0.001 | 0.98 (0.77–1.24) | 0.84 |  |
| Mean instead of highest primary core | 730 (421) | 1.66 (1.32–2.10) | <0.001 | 0.98 (0.77–1.24) | 0.87 |  |

Margin (R), grade, lymphatic (L) and venous (V) invasion were not recorded for all patients, so these rows are complete-case fits. The last column gives the primary model, without the added covariates, refitted in the same patients; it shows how much of the change in each row reflects the smaller sample. Treatment era: year of operation 1996–2005, 2006–2012, 2013–2020. pM1: documented distant metastasis at resection; patients without a recorded M category were counted as not pM1. The accession-key row is a data check (two patients excluded).

**Table S4. TF grade within each clinicopathologic category (row percentages; 1,006 patients).** Each row gives the distribution of the three TF grades among the tumors in that category, complementing the column percentages of Table 1. Patients with unknown or not-assessable values are omitted.

| **Category** | **n** | **TF-negative** | **TF-low** | **TF-high** | **p** |
| --- | --- | --- | --- | --- | --- |
| **pT category** |  |  |  |  | <0.001 |
| T1 | 178 | 116 (65.2%) | 46 (25.8%) | 16 (9.0%) |  |
| T2 | 190 | 107 (56.3%) | 47 (24.7%) | 36 (18.9%) |  |
| T3 | 592 | 272 (45.9%) | 157 (26.5%) | 163 (27.5%) |  |
| T4 | 30 | 15 (50.0%) | 5 (16.7%) | 10 (33.3%) |  |
| **pN category** |  |  |  |  | <0.001 |
| N0 | 374 | 217 (58.0%) | 94 (25.1%) | 63 (16.8%) |  |
| N1 | 286 | 149 (52.1%) | 76 (26.6%) | 61 (21.3%) |  |
| N2 | 166 | 71 (42.8%) | 50 (30.1%) | 45 (27.1%) |  |
| N3 | 154 | 68 (44.2%) | 33 (21.4%) | 53 (34.4%) |  |
| **Neoadjuvant therapy** |  |  |  |  | 0.01 |
| Treatment-naïve | 294 | 152 (51.7%) | 88 (29.9%) | 54 (18.4%) |  |
| CROSS | 290 | 155 (53.4%) | 71 (24.5%) | 64 (22.1%) |  |
| FLOT | 83 | 37 (44.6%) | 15 (18.1%) | 31 (37.3%) |  |
| Other neoadjuvant | 181 | 98 (54.1%) | 42 (23.2%) | 41 (22.7%) |  |
| **Treatment era, all tumors** |  |  |  |  | 0.10 |
| 1996–2005 | 104 | 50 (48.1%) | 36 (34.6%) | 18 (17.3%) |  |
| 2006–2012 | 312 | 163 (52.2%) | 86 (27.6%) | 63 (20.2%) |  |
| 2013–2020 | 590 | 307 (52.0%) | 139 (23.6%) | 144 (24.4%) |  |
| **Treatment era, treatment-naïve tumors** |  |  |  |  | 0.74 |
| 1996–2005 | 76 | 36 (47.4%) | 27 (35.5%) | 13 (17.1%) |  |
| 2006–2012 | 95 | 52 (54.7%) | 27 (28.4%) | 16 (16.8%) |  |
| 2013–2020 | 123 | 64 (52.0%) | 34 (27.6%) | 25 (20.3%) |  |
| **Tumor regression (pretreated)** |  |  |  |  | 0.02 |
| >50% vital tumor | 281 | 140 (49.8%) | 59 (21.0%) | 82 (29.2%) |  |
| 10–50% vital tumor | 213 | 114 (53.5%) | 54 (25.4%) | 45 (21.1%) |  |
| <10% vital tumor | 69 | 44 (63.8%) | 17 (24.6%) | 8 (11.6%) |  |

P-values: χ² across the three TF grades (3 × k tables). For tumor regression, the χ² test for TF-high versus other tumors quoted in the main text gives p=0.004. Tumor regression is the proportion of vital tumor cells in pretreated resection specimens. Treatment era: year of operation.

**Table S5. TF expression in paired primary tumors and synchronous metastases.** Each patient contributes the highest-scoring assessable core of each compartment.

| **Measure** | **Lymph node, all** | **Lymph node, treatment-naïve** | **Lymph node, pretreated** | **Distant metastasis** |
| --- | --- | --- | --- | --- |
| Patients with both cores, n | 247 | 82 | 154 | 10 |
| Agreement, TF-positive (H > 10) | 72.9% | 73.2% | 74.7% | 40.0% |
| κ, TF-positive (95% CI) | 0.46 (0.34–0.57) | 0.45 (0.24–0.63) | 0.49 (0.35–0.62) | n/a |
| Agreement, TF-high (H > 100) | 83.4% | 84.1% | 83.1% | 80.0% |
| κ, TF-high (95% CI) | 0.57 (0.44–0.68) | 0.62 (0.43–0.80) | 0.55 (0.38–0.69) | n/a |
| Linearly weighted κ, three-tier grade (95% CI) | 0.51 (0.41–0.59) | 0.53 (0.38–0.67) | 0.52 (0.40–0.62) | n/a |
| TF-positive, primary vs metastasis (McNemar p) | 55.9% vs 51.4% (0.22) | 61.0% vs 56.1% (0.52) | 53.2% vs 50.0% (0.52) | 40.0% vs 60.0% (0.69) |
| TF-high, primary vs metastasis (McNemar p) | 25.1% vs 27.1% (0.53) | 26.8% vs 32.9% (0.27) | 25.3% vs 24.0% (0.85) | 30.0% vs 10.0% (0.50) |
| Median H-score, primary vs metastasis (Wilcoxon p) | 20 vs 20 (0.91) | 40 vs 28 (0.52) | 20 vs 10 (0.66) | 0 vs 45 |
| Spearman correlation of H-scores | 0.66 | 0.69 | 0.66 | 0.23 |

κ: Cohen’s kappa, unweighted for the two dichotomies and linearly weighted for the three-tier grade, with percentile bootstrap 95% CIs (10,000 resamples). McNemar: exact test. Wilcoxon: signed-rank test retaining tied pairs (Pratt method). The distant-metastasis pairs (n=10) are shown descriptively (n/a: not computed). The treatment-naïve and pretreated subsets exclude 11 patients with unknown neoadjuvant status.

**Table S6. TF-high after FLOT versus CROSS, and overall survival in pretreated patients.**

| **Analysis** | **Patients** | **Estimate (95% CI)** | **p** |
| --- | --- | --- | --- |
| **TF-high after FLOT vs CROSS (logistic regression)** |  |  |  |
| Crude | 373 | OR 2.11 (1.25–3.56) | 0.005 |
| Crude, complete cases | 297 | OR 2.35 (1.30–4.24) | 0.005 |
| + ypT and ypN | 297 | OR 2.26 (1.24–4.11) | 0.008 |
| + ypT, ypN and regression | 297 | OR 2.19 (1.20–4.00) | 0.01 |
| + ypT, ypN, regression and year | 297 | OR 1.95 (1.01–3.76) | 0.05 |
| All patients, + year of operation only | 373 | OR 1.78 (1.01–3.14) | 0.05 |
| Operated 2010 or later, crude | 308 | OR 2.14 (1.24–3.70) | 0.006 |
| Operated 2010 or later, + ypT, ypN and regression | 246 | OR 2.34 (1.25–4.39) | 0.008 |
| **Overall survival in pretreated patients (Cox regression)** |  |  |  |
| TF-high vs TF-negative, primary covariates | 419 | HR 1.50 (1.11–2.02) | 0.008 |
| TF-high vs TF-negative, + regression grade | 419 | HR 1.49 (1.10–2.01) | 0.009 |
| Regression grade (per step towards less vital tumor) | 419 | HR 0.93 (0.77–1.11) | 0.40 |

Upper block: odds ratios for TF-high expression after perioperative FLOT versus CROSS chemoradiotherapy (logistic regression). Complete cases are patients with known ypT, ypN and regression grade; 76 patients were excluded, 71 without a regression grade and 7 without ypT or ypN (some lacked both). Year of operation is entered per 5 years. Lower block: Cox models in the 419 of 517 pretreated patients with a regression grade (>50%, 10–50%, <10% vital tumor), adjusted for age, pT and nodal status.
